# Blood Based Biomarkers of Central Nervous System Involvement in Wilson’s Disease

**DOI:** 10.3390/diagnostics13091554

**Published:** 2023-04-26

**Authors:** Agnieszka Antos, Anna Członkowska, Jan Bembenek, Marta Skowronska, Iwona Kurkowska-Jastrzębska, Tomasz Litwin

**Affiliations:** 1 Second Department of Neurology, Institute of Psychiatry and Neurology, 9 Sobieskiego Str., 02-957 Warsaw, Poland; agantos@ipin.edu.pl (A.A.); czlonkow@ipin.edu.pl (A.C.); mskowronska@ipin.edu.pl (M.S.);; 2 Department of Clinical Neurophysiology, Institute of Psychiatry and Neurology, 02-957 Warsaw, Poland; jbembenek@ipin.edu.pl

**Keywords:** Wilson’s disease biomarkers, copper, exchangeable copper, magnetic resonance imaging, neurofilaments, glial fibrillary acidic protein, microtubule associated protein tau, ubiquitin C-terminal hydrolases

## Abstract

Wilson’s disease (WD) is an inherited disorder of copper metabolism with clinical symptoms related to pathological copper accumulation, which are mainly hepatic and/or neuropsychiatric. The disease is potentially treatable with pharmacological agents (chelators or zinc salts). As such, key factors for a favorable treatment outcome are early diagnosis and anti-copper treatment initiation as well as appropriate treatment monitoring for safety and efficacy. Despite the generally favorable outcome in most treated patients, almost 10% of the general population of WD patients and about 25% of patients in the group with initial neurological phenotype of disease experience early neurological deterioration. In almost 50% of patients with neurological symptoms, the symptoms persist. A search for new treatment modalities (e.g., gene therapy, molybdenum salts) aims to prevent early neurological deterioration as well as improve treatment outcomes. In addition to evaluating the clinical signs and symptoms of the disease, serum biomarkers for diagnosis and treatment monitoring are very important for WD management. Sensitive serum biomarkers of copper metabolism and liver injury are well described. However, there is a need to establish blood-based biomarkers of central nervous system (CNS) injury to help identify patients at risk of early neurological deterioration and aid in their monitoring. Based on the available literature and studies of WD patients, the authors reviewed serum biomarkers of CNS involvement in WD, as well as their potential clinical significance.

## 1. Introduction

Wilson’s disease (WD) is an inherited disorder of copper metabolism with pathological copper accumulation in different organs, particularly the liver and brain, leading to their damage and consecutive clinical symptoms [1,2,3]. WD generally manifests between early childhood and late adulthood and is associated with a wide spectrum of clinical symptoms, from asymptomatic cases to advanced liver disease, acute liver failure, and/or severe neurological symptoms including dystonia, tremor, and chorea, among others [1,2,3]. There appear to be no clear genotype-phenotype correlations or well-established factors linked to clinical presentation [2].

Importantly, WD can be successfully treated with pharmacological agents including copper chelators or zinc salts that inhibit copper absorption from the digestive tract. However, treatment must be introduced early, before irreversible changes in the liver and brain occur [1,4,5,6,7,8,9]. Following treatment initiation, the patient should be monitored for adverse events (some may be severe), compliance (which is often problematic), as well as efficacy [1,4,5,6,7,8,9].

Another important problem is early (paradoxical) neurological deterioration, which is often irreversible and may occur early (up to 6 months) after initiation of anti-copper treatment [3,4,10,11]. Rapid mobilization of copper from tissues and a transient increase in toxic “free” copper in the blood has been suggested to be a potential mechanism, especially following initiation of chelators such as d-penicillamine (DPA) [1]. Although dose titration (“start low and go slow”) has reduced its occurrence, up to approximately 11% of all WD patients and as many as 25% of neurological cases experience neurological deterioration [4,5,10,11,12]. These therapeutic issues have resulted in investigations into new treatment modalities, such as gene therapy, molybdenum salts, methanobactin, etc., which aim to be more effective, safe, and useful for preventing early neurological deterioration [8,12].

WD management, especially treatment monitoring, includes detailed clinical and laboratory analysis focused on copper metabolism, worsening or improvement of clinical signs and symptoms, e.g., using scales like the Unified Wilson’s Disease Rating Scale (UWDRS) or the Global Assessment Scale for WD (GAS for WD) for neurological symptoms [13,14], and the Model of End Stage of Liver Disease (MELD), Nazer Score and New Wilson Index for liver failure and fibrosis assessment (the Fibrosis-4 (*FIB*-*4*) Index for Liver Fibrosis) [1,2,6,8,9]. Additionally, structural assessments are performed, such as using a brain magnetic resonance imaging (MRI) semiquantitative scale to assess acute toxicity or chronic damage, [15,16] or using ultrasound examination, computed tomography (CT), MRI with or without elastography, and fibroscan for liver fibrosis assessment [1,8,15,16]. In addition, there is a need to find quantitative and objective diagnostic and therapeutic blood-based biomarkers of central nervous system (CNS) injury to: (1) facilitate treatment monitoring; (2) identify patients who are at risk of neurological deterioration; and (3) aid in the investigation of disease progression, especially neurological disease progression, to observe the natural course of disease [11,12,15,16,17]. The aim of this review is to provide an overview of serum biomarkers of CNS injury that have been assessed in WD.

## 2. Proposed Blood-Based Biomarkers of CNS Involvement in WD

### 2.1. Serum Protein Biomarkers

#### 2.1.1. Neurofilament Light Chain

Neurofilament proteins (NfPs) maintain the structural integrity of the neuronal cytoskeleton and consist of five subunits: neurofilament light chains (NfL), neurofilament medium chains (NfM), neurofilament heavy chains (NfH), alpha-internexin (INA), and peripherin (PRPH) [18,19,20,21,22,23,24,25,26,27,28]. In pathological conditions affecting the CNS, these proteins are released in large amounts from neurons into the cerebrospinal fluid (CSF) and blood, and they are sensitive fluid biomarkers of neuroaxonal injury [19,20,21,22]. Such processes also occur in physiological conditions during brain development and aging, but to a much lesser degree. Observations during aging have shown a 3.3% per year increase in levels of serum NfL (sNfL), which may also be affected by sex, since men have higher concentrations, potentially due to the neuroprotective effect of estrogens in women [19,20]. High NfL levels in serum and CSF have been detected in several neurodegenerative and neuroinflammatory disorders, including Parkinson’s disease (PD), Alzheimer’s disease (AD), Huntington disease, multiple sclerosis (MS) and meningitis, which correlated with the severity and progress of disease, brain and spine magnetic resonance pathological changes (especially atrophy), and “new” demyelinating lesions in MS [18,19,20,21,22,28]. Further, elevated sNfL levels were described as valuable in the differential diagnosis of PD and PD plus syndromes [18,19,20,21,22]. Importantly, NfL can be measured in CSF as well as in serum, being about 40-fold higher in CSF, but comparative studies in neurodegenerative disorders indicate concordance, and analysis from serum is less invasive than from lumbar puncture [18]. As such, sNfL are currently used as an “analogue of C-reactive protein in neurologic disease” as a universal biomarker of brain injury in clinical trials and experimental studies [6,21,28].

Five papers analyzing sNfL in WD have been published so far, all in the last 2 years [23,24,25,26,27]. The first study assessing the significance of sNfL was performed by Shribmann et al. in 2021 [23]. They analyzed plasma samples from 40 WD patients (23 with neurological phenotype and 17 with hepatic) and 38 age-matched controls. sNfL concentrations were significantly higher in neurological patients (8.7 ng/L) than in patients with hepatic manifestations (7.0 ng/L) as well as in healthy controls (7.6 ng/L) (*p* < 0.01). Interestingly, there were no differences in sNfL between hepatic patients and controls. Further, a group of patients with high activity of disease (HAD) was established who had either progressed from hepatic symptoms alone to the neurological form or who had the neurological form and worsened during observation. Patients with HAD had particularly high sNfL concentrations (22.2 ng/L) compared with the other patients (7.7 ng/L; *p* < 0.01). Additionally, the highest sNfL concentrations were observed in patients whose neurological symptoms had paradoxically worsened (38.5 ng/L) [23]. Finally, the authors using receiver operating characteristic (ROC) curves to assess diagnostic performance in differentiating groups (neurological WD, hepatic WD, and control group), calculated sensitivity and sNfL cut-offs values at 70%, 80%, and 90% specificities, where the area under the curve (AUC) was increased (*p* < 0.05). Results AUC for NfL in differentiating neurological and hepatic WD patients was 0.707 (*p* < 0.05) and for 70%, 80%, and 90% specificities, the sensitivities were 61%, 48%, and 39% with sNfL cut-off values 8.4, 9.7, and 12.9 ng/L, respectively [23]. These observations suggested that sNfL could be a sensitive fluid biomarker of CNS involvement in WD and encouraged further studies [23].

The next two studies documenting the significance of sNfL in WD were performed by Ziemssen and colleagues [26,27]. The first study analyzed 61 newly diagnosed, drug-naive patients (36 neurological, 18 hepatic and 7 asymptomatic) and confirmed that patients with the neurological phenotype had significantly higher sNfL concentrations than hepatic and presymptomatic cases (38.7 vs 13.3 ng/mL; *p* < 0.01). Additionally, patients with more severe neurological forms of the disease tended to have higher mean sNfL levels than the less severe tremor form (dystonia, 78.7 ng/mL; parkinsonism, 44.9 ng/mL; tremor, 16.2 ng/mL; *p* = 0.065). The authors additionally documented positive correlations between sNfL concentrations and UWDRS part II and III scores (r = 0.37 and 0.38; *p* < 0.05) as well as semiquantitative brain MRI scale scores for acute toxicity (r = 0.48; *p* < 0.01) and the chronic damage score (r = 0.54; *p* < 0.01). Therefore, using additional tools, the previous observations by Shribman et al. [23] were confirmed regarding the significance of sNfL as a reliable and sensitive biomarker of CNS injury in WD. In another study [27], the same researchers, analyzing various risk factors of early neurological deterioration (apart from the clinical factors, initial severity of neurological disease scored in UWDRS and the chronic damage score in brain MRI) found that initial sNfL concentrations, prior to anti-copper treatment introduction, could be a risk factor for early neurological deterioration [27]. Initial sNfL concentrations in the group of patients who neurologically deteriorated were 33.2 compared with 27.2 ng/mL (*p* < 0.01) in patients who did not [27]. In further detailed results of ROC analyses of early neurological deterioration, sNfL were characterized by AUC 0.770, with optimal cut-off of 18.15 ng/mL, sensitivity of 80%, specificity of 72.5% with a positive predictive value (PPV) of 0.363 and a negative predictive value (NPV) of 0.95 [27]. Additionally, authors performed the bivariate logistic models, and when corrected for baseline UWDRS part III scores (odds ratio (OR) = 7.14), only sNfL remained as a significant predictor of neurological worsening (OR = 6.94 [27]). These results emphasize the significance and potential usefulness of sNfL as a sensitive biomarker of neurological injury in WD.

These studies from Europe (United Kingdom, Germany, and Poland) were further confirmed by researchers from China [24,25]. In 2022, Yang et al. [25] analyzed 75 WD patients (54 neurological and 21 hepatic) and 27 age-matched healthy controls, and similarly found higher sNfL concentrations in neurological WD patients than in hepatic (8.1 vs. 3.1 pg/mL; *p* < 0.01), with no difference between hepatic WD and controls and a positive correlation between sNfL and UWDRS (r = 0.29; *p* < 0.05). Additionally, they found significant negative correlations between sNfL concentrations and brain atrophy volumes of total gray matter, caudate nucleus, putamen and nucleus accumbens analyzed via brain MRI with FreeSurfer and voxel-based morphometry (*p* < 0.01). Using ROC curves, a cut-off value of 5.51 pg/mL was able to distinguish between hepatic and neurological forms with 85.2% sensitivity and 90.5% specificity. They also confirmed that patients who were neurologically unstable (drug naïve, neurologically deteriorated, or hepatic patients with neurological symptoms) had higher sNfL concentrations than stable patients (10.7 vs. 7.2 pg/mL; *p* < 0.01).

Based on these promising results, Wang et al. [24] from China performed a pilot study to further verify the significance of sNfL assessment using longitudinal observations. Initially, they analyzed 186 WD patients and 77 controls and again found higher sNfL concentrations in neurological patients than in hepatic, with a lack of difference between hepatic and healthy control groups, and positive correlations between sNfL and UWDRS total scores (r = 0.47; *p* < 0.01, *n* = 107) as well as with the brain MRI semiquantitative total scale (r = 0.49; *p* < 0.01, *n* = 114). Additionally, they investigated the effect of anti-copper treatment on sNfL concentrations by analyzing 34 WD patients with median follow-up of 1251 days. No correlations between UWDRS and sNfL concentrations were found at the follow-up visit. Additionally, when the 34 WD patients were divided into three groups based on UWDRS score severity, there were no significant correlations between clinical improvement, stabilization, or deterioration and sNfL. However, this was a very small group of patients (34 divided into three groups) and longitudinal observations in WD according to sNfL significance should be performed on a much larger group of heterogenous WD patients.

Taking these results together, sNfL appears to be a useful complementary tool to add to clinical and brain MRI assessments for WD treatment monitoring [28].

#### 2.1.2. Glial Fibrillary Acidic Protein

Glial fibrillary acid protein (GFAP) is found in intermediate filaments, which form the astrocyte cytoskeleton, and is often used by researchers as a marker of astrocyte damage [29,30]. In addition to astrocytes, GFAP is found in ependymal cells, retinal Muller cells, and nonglial tissues, such as hepatic stellate cells, which can be transformed into cells with myofibroblast phenotype during liver injury and play a key role in liver fibrosis (cirrhosis). Furthermore, GFAP has rarely been detected in myoepithelial cells, some chondrocytes, and in some tumors (e.g., papillary meningiomas, salivary glands tumors, and cartilaginous tumors) [29,30]. It serves as a marker of CNS damage, reflecting the proliferation and hypertrophy of astrocytes (with increase GFAP synthesis) in the course of CNS injury [29,30].

GFAP levels are increased and associated with the severity of neuroimaging-based diagnosed head injury, with high levels predictive of poor outcome and negatively correlated with the Glasgow Coma Scale [29]. In addition, in neuroinflammatory disorders such as MS, positive correlations between serum GFAP concentrations and disease progression using clinical scales have been observed [29,30].

In WD, both pathological forms of astroglia (Alzheimer type 1 and 2 cells and Opalski cells) as well as astroglial cell proliferation have been described in neuropathological studies, and in hepatic encephalopathy, the proliferation and role of CNS astrocytes is crucial [1,3,8]. These findings provide strong scientific basis for GFAP as a marker of CNS involvement. However, to date, only two studies have analyzed its significance in WD. Shribman et al. [23] did not find a significant difference in mean GFAP levels between WD patients (84 ng/L; *n* = 40) and healthy controls (84 ng/mL; *n* = 38) or by clinical phenotype (neurological, 84 ng/L; hepatic, 80 ng/L).

The second study, performed by Lin et al. [29], included a larger population of 94 patients (74 neurological and 20 hepatic) and 25 healthy controls. Serum GFAP concentrations were higher in patients with the neurological phenotype (143.8 pg/mL) than the hepatic form (107.5 pg/mL) and controls (86.8 pg/mL) (*p* < 0.01). However, there were no statistically significant differences between hepatic patients and healthy controls. The presence of neurological symptoms was found to be the only independent factor affecting GFAP concentrations. ROC curves analysis showed that a cut-off value of 128.8 pg/mL could distinguish neurological and hepatic WD patients (sensitivity 80% and specificity 63.5%). There were no correlations between serum GFAP and WD severity scored using UWDRS or the brain MRI semiquantitative scale for WD [29].

Summarizing the available results, conflicting data as well as a lack of correlation with neurological severity appear to reduce the significance of current studies with serum GFAP as a biomarker of neurological CNS involvement in WD [23,29]. Further studies with larger populations and more patients with severe neurological symptoms are needed to establish serum GFAP as a biomarker in WD [30].

#### 2.1.3. Tau Protein

Tau protein belongs to the family of microtubule-associated proteins (MAPs) essential for stabilization of microtubules, binding them to neurofilaments and stabilizing the cytoskeleton [31,32,33,34]. In generally healthy conditions, normal tau is found in axons, while in neurodegenerative disorders, it is translocated to neurons and dendrites, forming abnormal depositions of excessive phosphorylated tau proteins [31,32,33,34].

Importantly, in the etiology of neurodegenerative disorders (the “taupathies”), the affinity of tau proteins for microtubules depends on the degree of their phosphorylation—higher levels of tau protein phosphorylation lead to filamentous degeneration and pathological tau aggregations as neurofibrillary tangles, which correlate with the severity of disorders such as AD [31,32,33,34]. Several other factors, such as corticosteroids, stress, toxins, and neuroinflammation may also lead indirectly to excessive phosphorylation of tau proteins [34]. In addition to neurodegenerative disorders, high serum and CSF tau levels have been observed after brain injuries, in acute stroke, and after epileptic seizures [32].

Two studies analyzing tau protein in WD have been performed to date. In 2019, Lekomtseva et al. [35] analyzed 47 WD patients (all neurological phenotype, 19 additionally had symptomatic liver disease) and 30 healthy controls. Increased mean serum tau concentrations were seen in WD patients compared with controls (221.1 pg/mL vs. 71.1 pg/mL; *p* < 0.01). No other correlations were found between serum tau protein levels and copper metabolism or duration of disease, and researchers did not assess any possible associations with severity of neurological disease. In the study by Shribman et al. [23], there was no difference in serum tau concentrations between WD patients and controls (1.4 ng/L hepatic, 1.8 ng/L neurological and 1.4 ng/L controls). However, positive correlations between serum tau levels and UWDRS (part II beta = 0.06; *p* < 0.01; and part III beta = 0.06; *p* < 0.01) were observed. Further studies with larger numbers of WD patients with different phenotypes and varying degrees of neurological symptoms are needed to verify the possible role of tau proteins as a biomarker in WD.

#### 2.1.4. Ubiquitin Carboxyl-Terminal Hydrolase L1

Up to 5% of the total protein in the brain is ubiquitin carboxyl-terminal hydrolase L1 (UCH-L1) [31,32]. It hydrolyses small C-terminal adducts of ubiquitin to produce ubiquitin monomer, which is needed for axonal stability. UCH-L1 expression is mostly seen on neurons, cells of the neuroendocrine system, testes, and ovaries [17,23,31,32]. Mutations in *UCH-L1* gene are described in the etiology of PD and AD as causing disturbances with other proteins involved in neurodegenerative disorders (parkin and alfa synuclein) [23]. Only one investigation, the study by Shribman et al., has assessed UCH-L1 in patients with WD [23]. They found increased serum UCH-L1 concentrations in WD patients with the neurological phenotype compared with healthy controls (33 ng/L vs. 23 ng/L; *p* < 0.05). There was no difference between neurological and hepatic WD patients, or between hepatic and healthy controls. Further positive correlations between serum tau levels and UWDRS (part II beta = 2.7; *p* < 0.01; and part III beta = 0.95; *p* < 0.01) were observed. Further studies using UCH-L1 as a potential biomarker in WD are needed to verify its significance.

### 2.2. Serum Copper Metabolism Biomarkers

The classical biomarkers of copper metabolism (serum ceruloplasmin and copper levels, and daily urinary copper excretion) are used to establish the WD diagnosis as well as for monitoring, as described in international guidelines [1,3,36]. Copper-related biomarkers, particularly daily urinary copper excretion, are included in the Leipzig score for WD diagnosis as well as in recommendations for WD treatment. To improve diagnosis as well as treatment monitoring of WD, several additional copper metabolism biomarkers were studied and still are under investigation, such as non-ceruloplasmin bound copper (NCC), exchangeable copper (CuEXC), relative exchange copper (REC), albumin-copper (Cu-ALB), directly measured non-ceruloplasmin bound copper (dNCC), and labile bound copper (LBC) [17,37]. However, in this review, we only presented three copper-based biomarkers that have been found to be related to CNS involvement: NCC [1,35,36], CuEXC [37] and REC [1,37].

#### 2.2.1. Non-Ceruloplasmin-Bound Copper

NCC is currently the most evaluated endpoint in WD studies, as it theoretically reflects the toxic fraction of copper: so-called free-copper [1]. Current studies evaluated it indirectly by multiplying the ceruloplasmin levels (measured with nephelometry, measured in mg/dL) 3.14 times and then subtracting this fraction from serum copper levels (expressed in µg/dL). In healthy individuals and adequately treated WD patients, serum NCC are in the range of 5–15 µg/dL [1]. In drug-naïve patients or just after anti-copper treatment initiation, NCC is usually >15 µg/dL [1]. However, the current method used for NCC calculation is indirect, non-validated, and may give non-diagnostic false negative results in up to 20% of patients, particularly when using immunological methods of ceruloplasmin assessment. Direct methods to measure dNCC are very promising and under investigation [1,17].

Only a small number of studies have analyzed correlations between NCC and CNS injury, likely due to the methodological problems described above. Smolinski et al. [36] found that NCC concentration was negatively correlated with WD severity, based on both UWDRS part II and UWDRS part III (r = −0.295, *p* < 0.05 and r = −0.315, *p* < 0.05, respectively; age- and sex-adjusted r = −0.318, *p* < 0.05 and r = −0.382, *p* < 0.05, respectively). They also found that the NCC concentration was significantly associated with brain atrophy when analyzed with brain MRI, based on brain parenchyma fraction (r = −0.389, *p* < 0.01) and the ventricular CSF volume (r = 0.420, *p* < 0.01). In another study, Redzia-Ogrodnik et al. [16] analyzed 100 WD patients and found positive correlations between NCC and acute toxicity score from the brain semiquantitative scale for WD (r = 0.19; *p* < 0.05). Given the correlations seen, further studies with directly measured NCC will be the key to clearly establishing its role in the analysis of severity of CNS involvement [9,12].

#### 2.2.2. Exchangeable Copper

Exchangeable copper (CuEXC) reflects the labile fraction of copper bound to albumins and other peptides in serum, which can easily be exchanged in the presence of a high-copper-affinity chelating agent (ethylenediaminetetraacetic acid [EDTA]) [1,37]. This fraction can be assessed directly, but this analysis requires specific pre-analytical conditions [37]. Blood samples collected for CuEXC must be very quickly (up to 30 min centrifugated with serum) taken and frozen until analysis, a task which must be performed within 7 days [37], an additional factor that limited use of this biomarker [17,31]. The use of CuEXC in WD diagnosis has been described in several papers and studies from France. However, to date, it has not been included in clinical recommendations apart from in France [1,37,38,39,40]. To assess the use of CuEXC as a biomarker of neurological injury, Poujois et al. [37] evaluated 48 newly diagnosed WD patients, distinguishing WD phenotypes as hepatic, extra-hepatic (mainly neurological), or presymptomatic. Patients with extrahepatic manifestation of WD presented with significantly higher CuEXC values than those with the hepatic form (2.75 µmol/L vs. 1.26 µmol/L; *p* < 0.01). Performing ROC curves, the cut-off for extrahepatic (neurological) presentation of WD was 2.08 µmol/L (sensitivity 85.7%; specificity 94.1%). Additionally, CuEXC positively correlated with the UWDRS (r = 0.45, *p* < 0.05) and the brain WD MRI score (r = 0.38, *p* < 0.05).

The authors concluded that in addition to its utility in WD diagnosis, CuEXC could be used as a biomarker of CNS involvement in WD (and severity). However, as this methodology is only currently used in France, their findings should be replicated in international studies and larger cohorts of WD patients.

Another parameter of copper metabolism (used mainly in France), relative exchange copper (REC), which reflects percentage of CuEXC to total serum copper, is very promising as highly accurate tool, especially in WD diagnosis. In differential WD diagnosis (WD patients, heterozygous carriers, and healthy control) a cut-off of 18.5% was shown to be more sensitive and specific (100% both) than other copper metabolism tests in WD diagnosis [37]. However, Poujois, et al. [37], analyzing CuEXC, REC and severity of neurological WD, did not find statistically significant differences in REC values between hepatic and extrahepatic patients (hepatic: 27% vs extrahepatic: 40.5%; *p* = 0.09). That is why the significance of REC as biomarker of neurological injury in WD is currently limited [17,37].

## 3. Conclusions

Results from this review indicate that there are many interesting potential blood-based biomarkers of CNS involvement in WD that should be further evaluated, particularly in larger and more heterogenous (according to clinical symptoms) groups of WD patients. sNfL is the most-studied objective serum biomarker of severity of neurological disease in WD, which could and should be used as complementary tool with clinical (UWDRS) and neuroradiological assessment (brain MRI scale), since sNfL is also likely a risk factor for neurological deterioration in drug-naïve patients [23,24,25,26,27]. Wang et al. [24] did not find associations with NfL in longitudinal studies, which was likely due to low patient numbers and the low severity of neurological disease in the included patients. Further larger studies are warranted.

GFAP, tau protein, and UCH-L1 have good scientific background as biomarkers of CNS involvement in WD, and again, further studies should be performed in larger populations of WD patients [23,29,35]. Direct measurement methods (dNCC, LBC) should be used in additional studies of NCC as a copper biomarker, while CuEXC needs to be validated in international studies [37,39]. It is important that these studies are performed to meet the need for reliable, objective blood-based biomarkers of CNS involvement in order to quantitatively assess the neurodegenerative process and understand its natural course [41,42]. This will aid in predicting early neurological deterioration [27] and other neurological outcomes, refining treatment monitoring, and developing new treatments.

## Data Availability

Not applicable.
